# Transfer Learning on Small Datasets for Improved Fall Detection

**DOI:** 10.3390/s23031105

**Published:** 2023-01-18

**Authors:** Nader Maray, Anne Hee Ngu, Jianyuan Ni, Minakshi Debnath, Lu Wang

**Affiliations:** Department of Computer Science, Texas State University, San Marcos, TX 78666, USA

**Keywords:** fall detection, transfer learning, small dataset

## Abstract

Falls in the elderly are associated with significant morbidity and mortality. While numerous fall detection devices incorporating AI and machine learning algorithms have been developed, no known smartwatch-based system has been used successfully in real-time to detect falls for elderly persons. We have developed and deployed a SmartFall system on a commodity-based smartwatch which has been trialled by nine elderly participants. The system, while being usable and welcomed by the participants in our trials, has two serious limitations. The first limitation is the inability to collect a large amount of personalized data for training. When the fall detection model, which is trained with insufficient data, is used in the real world, it generates a large amount of false positives. The second limitation is the model drift problem. This means an accurate model trained using data collected with a specific device performs sub-par when used in another device. Therefore, building one model for each type of device/watch is not a scalable approach for developing smartwatch-based fall detection system. To tackle those issues, we first collected three datasets including accelerometer data for fall detection problem from different devices: the Microsoft watch (MSBAND), the Huawei watch, and the meta-sensor device. After that, a transfer learning strategy was applied to first explore the use of transfer learning to overcome the small dataset training problem for fall detection. We also demonstrated the use of transfer learning to generalize the model across the heterogeneous devices. Our preliminary experiments demonstrate the effectiveness of transfer learning for improving fall detection, achieving an F1 score higher by over 10% on average, an AUC higher by over 0.15 on average, and a smaller false positive prediction rate than the non-transfer learning approach across various datasets collected using different devices with different hardware specifications.

## 1. Introduction

**Figure 1:**
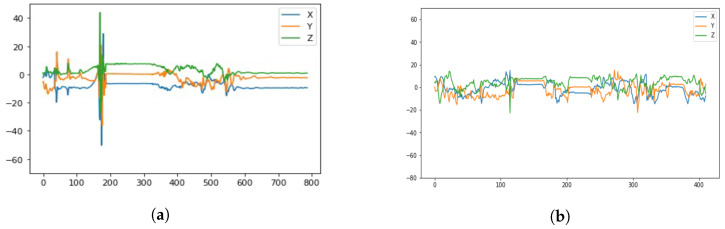
Two different smartwatch accelerometer data. (**a**) Acceleration from a fall. (**b**) Acceleration from putting on a jacket.

Falls are one of the leading causes of death and injury among the elderly population [1]. According to the U.S. Center of Disease Control and Prevention, one in four Americans aged 65 and older falls each year [2]. A recent CDC report also stated that around 28% of people aged over 65 lived alone [3]. In addition, the Agency for Healthcare Research and Quality reports that each year, somewhere between 700,000 and 1,000,000 people in the United States fall in the hospital alone [4]. The resultant inactivity caused by a fall in older adults often leads to social isolation and increased illnesses associated with inactivity including infections and deep vein thrombosis. Consequently, a large variety of wearable devices which incorporate fall detection systems have been developed [5,6,7,8]. Wearable devices have the promise of bringing personalized health monitoring closer to the consumers. This phenomenon is evidenced in the articles entitled “Staying Connected is Crucial to Staying Healthy” (WSJ, 25 June 2015) and “Digital Cures For Senior Loneliness” (WSJ, 23 February 2019). The popularity of using a smartwatch, paired with a smartphone, as a viable platform for deploying digital health applications is further supported by release of the Apple Series brand of smartwatches [9] which has a built-in “hard fall” detection application as well as an ECG monitoring App. Apple also added car crash detection in the most recently version of Apple watches. An Android-Wear based commercial fall detection application called RightMinder [10] has been released on Google Play since 2018. One of the major sensors used in fall detection on a smartwatch is an accelerometer, which measures the acceleration of an object. Acceleration is the change in velocity with respect to time and velocity represents the rate at which an object changes its position. Acceleration data is commonly used in fall detection because accelerometer sensors are found in most smart devices, and a distinct change in acceleration happens when a fall occurs. The clustered spikes in Figure 1a show a unique pattern in the acceleration data during one second when the fall occurs, which means that falls can be identified in acceleration data by that pattern.

Previously, we have developed a watch-based SmartFall App using long short-term memory neural networks (LSTM), an artificial recurrent neural network (RNN) with feedback connections, to detect falls based on the above pattern, by training it on simulated fall data collected using a Microsoft watch (MSBAND) [11,12]. We have deployed this SmartFall system on a commodity-based smartwatch which has been trialled by nine senior participants. Each participant was recruited under IRB 7846 at Texas State University to use the SmartFall system to collect their ADLs (activity of daily living) data by just asking them to wear the watch for three hours per day over a seven day period. The user only needs to interact with the watch and provide feedback when false positives are generated by the system. Despite the system was welcomed by the participants in our trials, it still have several limitations: (1) fall detection models trained on simulated falls and ADLs performed by young, healthy test subjects suffer from the fact that they do not exhibit the same movement characteristics as the elderly population. For example, an elderly person typically has comorbidities that affect their movements including the effects of multiple medications, poor vision, stroke, arthritis, sensory neuropathies and neuro-degenerative diseases such as Parkinson’s disease, all of which may contribute to their risk of falling [13]; (2) a sudden hand or wrist movement from some ADLs can interfere with the recognition of this pattern. For example, Figure 1b is the signal generated from a person putting on a jacket and has some cluster spikes which can be mistaken for a fall; (3) there is no guarantee that accelerometer data collected from different smartwatch devices is exactly of the same quality for fall detection since they have different hardware characteristics and API libraries.

In addition, we find that a fall detection model trained with data collected using a specific device usually does not generalize well to similar data collected using a different device because of differences in hardware characteristics which result in the acceleration data being sensed and recorded with varying G units, sampling rates, and X, Y and Z orientations of the accelerometer data. For example, Huawei watch specified that data can be collected in 32 ms, but in reality, the data is always collected in every 20 ms while MSBAND collects data in 32 ms as specified. To tackle the aforementioned issues, we propose to use transfer learning approach to solve the small dataset problem in smartwatch based fall detection system. More specifically, while collecting a large amount of ADL or fall data from the elderly population is an unrealistic task (i.e., the target domain), collecting a small amount of everyday movement data from the elderly population is possible (i.e., the source domain). Therefore, the obtained model in the source domain can be utilized and retrained in the target domain. This will enable us to create a real-world smartwatch-based fall detection model usable by older adult where we only need to collect a small amount of data to train a model tailored to each of them.

In this paper, we first demonstrate that transfer learning is an effective strategy for overcoming the small data set problem in fall detection by using data collected from the same type of device (meta-sensor) on both left and right wrists. After that, we leverage the pretrained model on one device and generalize the model via transfer learning on another device. For instance, we perform a set of experiments that transfer a LSTM fall detection model that we published in [11] using data collected with the source MSBAND device to a target meta-sensor device. We show that the fall detection model created via transfer learning has a higher F1-score than the LSTM model created directly from the limited meta-sensor data trained from scratch. We also demonstrate that another small fall data set collected using a Huawei smartwatch performed better when trained using transfer learning from a pretrained MSBAND model as well. Finally, we show that fall detection can be improved by enabling a scablable way to add new sensors to improve our fall detection system via training an ensemble of classifiers using transfer learning. For example, adding accelerometer data sensed from a cell phone might resolve the false positives generated from an ADL shown in Figure 1b. The main contributions of this paper are:

- Collecting three datasets including accelerometer data for fall detection problem from different devices: the MSBAND watch, the Huawei watch, and the meta-sensor device.
- Conducting an in-depth study of the effectiveness of transfer learning for fall detection using a small data set by creating effective left and right wrist fall detection models.
- Exploring the practicality of applying transfer learning on heterogeneous sensing devices by transferring an existing fall detection model, trained on our MSBAND data set, to a meta-sensor device (in one experiment), as well as a Huawei smartwatch (in another separate experiment), both using a small amount of device specific data.
- Demonstrating the improvement of fall detection using transfer learning to create an ensemble model of both left and right wrists or any additional heterogeneous sensing device.

The remainder of this paper is organized as follows. Section 2 describes the related work. In Section 3, we discuss the architecture of the SmartFall system and the App used for running the fall detection model created by the transfer learning. In Section 4, we provide the methodology used in establishing our hypothesis. This includes the detailed descriptions of how to collect three datasets from three different devices, the proposed LSTM model architecture, the tuning of hyperparameters of the LSTM model, and the transfer learning framework that was used for our experiments. In Section 5, the experimental procedures and results are described and shown. Finally, Section 6 concludes the paper.

## 2. Related Work

We firstly review the traditional healthcare area where transfer learning is intensively explored and then we conduct an overview of transfer learning methods with a focus on time-series data. Finally, we compare our method to the existing works which related to fall detection area, and clarify its novelties.

### 2.1. Transfer Learning for General Healthcare

Despite deep learning (DL) has achieved extraordinary success in a variety of tasks recently [14,15,16], one of the main drawbacks is DL usually relies on abundant labeled training examples. In many scenarios, collecting sufficient training data is time-consuming or even impossible. Semi-supervised learning method can address this problem by some extent since it only requires a limited amount of labeled data [17]. However, it fails to produce satisfactory models when unlabeled instances are difficult to obtain as well. Consequently, transfer learning, which emphasizes transferring knowledge between various domains, is a promising approach to address the aforementioned problem. More specifically, transfer learning aims to transfer the prior knowledge from existing domains to a new domain [18]. Currently, transfer learning can be divided into two categories due to the discrepancy between domains: homogeneous and heterogeneous transfer learning. In general, homogeneous transfer learning approaches try to deal with situations where the domains have the same feature space. In contrast, heterogeneous transfer learning methods are proposed to handle the situations where the domains have mismatched feature spaces [19].

Due to the fact that data collection is hard to conduct in the privacy-sensitive healthcare area, extensive studies have been proposed to adopt homogeneous transfer learning to solve the data scarcity issue [20,21,22,23,24]. For instance, Maqsood et al. [20] adopted and finetuned the AlexNet [15] for the Alzheimer’s disease detection problem. Initially, the AlexNet network is pretrained over an ImageNet [25] dataset (i.e., the source domain) first. After that, the convolutional layers of AlexNet are fixed, and the last three fully connected layers are replaced by one softmax layer, one fully connected layer, and one output layer. The modified AlexNet is then finetuned on the the Alzheimer’s data set [26] (i.e., the target domain). Results indicate that the proposed transfer learning approach retains the highest accuracy for this multiclass classification problem. Similarly, Shin et al. [21] applied the transfer learning method and fine-tuned the pre-trained convolutional neural networks (CNN) to solve the computer-aided detection problems. Moreover, Donahue et al. [27] proved that AlexNet [15] could improve the performances of various problems, including object recognition and scene recognition.

In addition to the aforementioned homogeneous transfer learning methods, heterogeneous transfer learning methods have been explored in healthcare area as well [28,29,30]. For example, Palanisam et al. demonstrated that by applying transfer learning method, model pretrained on image data, such as ImageNet [25], can recognize features on non-image data such as audio [28]. Specifically, the audio data was converted into spectrogram images first, and the knowledge from model which pretrained on ImageNet data can transfer to the spectrogram domain for audio classification problem. In addition, Koike et al. applied transfer learning method on the heart disease prediction from heart sounds [29]. They compared two transfer learning scenarios which pretrained on audio and image datasets, respectively, and highlight how models pretrained on audio can outperform the one from image models. In summary, it can be noted that all aforementioned works are based on the pretrained models on a large-scale source domain, such as ImageNet [25] dataset.

### 2.2. Transfer Learning for Time-Series Data

Time-series data has received huge attention due to its robustness against various viewpoints or illumination conditions [31,32]. In the healthcare domain, time-series data is also one of the most common types of data. However, transfer learning techniques for time-series data have been less evaluated [33,34,35,36,37,38] due to the absence of a large-scale accurately labeled dataset such as ImageNet [25] and the scarcity of publicly available time-series data in the healthcare domain. For instance, Li et al. [33] developed a novel deep transfer learning technique for time-series data to use already-existing datasets to overcome the target domain’s data shortage problem. Initially, they trained a deep neural network (DNN) using a large number of time-series data collected from various application fields so that the general properties of time-series data can be learned by this DNN model. After that, they implemented the transfer learning process of this model to another DNN model which is designed to solve a specific target problem. More specifically, they used single-channel data to train their single-channel DNN for sensor modality classification. After that, they built a multichannel DNN [34] by fine-tuning the single-channel DNN for each channel on the target domain, and thus the final multichannel DNN can recognize the outputs from all channels on the target domain. They evaluated their approach for human activity recognition (HAR) and emotion recognition (ER), and the results confirmed that the transfer learning strategy performs better than the baseline for both HAR and ER problems. Similarly, Gikunda et al. [35] adopted transfer learning as well as active learning to address this same problem of insufficiency of labeled time-series data. Results indicated that using only 20% of the training data, they achieved higher accuracy with hybrid transfer active learning than with existing techniques. More recently, Zhou et al. [37] proposed a novel dynamic transfer learning-based time-series prediction to address the issue of small datasets in industrial production. The proposed dynamic transfer learning framework was created using two features: feature mapping, and network structure. Results showed that when compared to the approach without transfer learning, the application of source domain knowledge can greatly improve target domain prediction performance in this dynamic transfer learning method. There are very few works that have explored transfer learning in a domain such as fall detection [38]. For example, Villar et al. [38] proposed a supervised fall detection model using online learning and transfer learning. They found that designing the fall detection specifically for each user rather than acquiring generalized models can lead to higher performance.

In summary, one of the common challenges that all of these previous works have faced is the scarcity of time series data and most of them implemented transfer learning to overcome this issue. However, none of them demonstrated the feasibility of transfer learning for overcoming the small data set problem in a real-world fall detection App. In addition, our study also explores the practicality of applying transfer learning on heterogeneous sensing devices using the same type of data collected from three different devices. This paves the way to overcome high false rates by placing other accelerometer sensors in different locations of the human body.

## 3. SmartFall System Architecture

We implemented a three-layered architecture which has the smartwatch on the edge, the smartphone in the middle layer, and the cloud server in the inner most layer. This is one of the most flexible architectures for IoT applications as discussed in [39] and is a practical choice for our prototype. Microservice is a particular implementation of the service-oriented architecture (SOA) that enables an independent, flexible, and distributed ways of deployment of services on the internet. Applications designed with microservices contain small, modular, and independent services which communicate via well-defined APIs. As compared to the three-layer architecture of our SmartFall, microservices are more agile, flexible, and resilient. However, each microservice must be hosted in a container and connected to a cloud framework. Moreover, the portability of an edge container is not proven yet. Currently, there are no Docker-compatible containers that can run on an edge device such as an Android phone. We have explored a microservice-based architecture called Accessor-based Cordova host for edge devices in [40].

**Figure 2:**
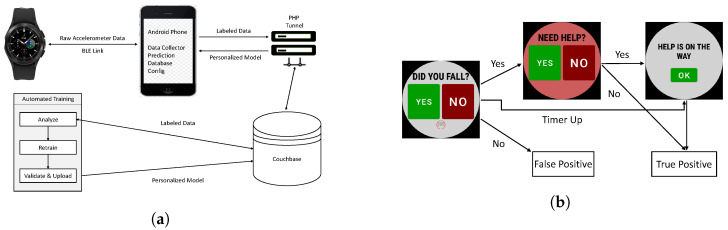
Architecture of SmartFall system. (**a**) An overview of the SmartFall system. (**b**) Watch’s user interface display after a fall is detected.

Figure 2a gives an overview of the SmartFall fall detection system. The major software components developed on a smartphone are (a) the *Config* module which manages the parameters, version of the deep learning model used by a particular user, the chosen personalization training strategy, and the chosen cloud server for data storage and re-training; (b) the *Database* module which manages all the data sensed, the uploading of the collected data to the cloud, and the downloading of the best re-trained model for a user; (c) the *Data Collector* module which manages the transfer of sensed data on the smartwatch to the smartphone using different communication protocols. Our smartwatch and smartphone currently communicate using BLE. The smartwatch and the server communicate using HTTP. Our system is designed to leverage multiple communication protocols; and (d) the *Prediction* module, which manages different machine learning models used for fall detection. For example, the system can be configured to run an ensemble recurrent neural network (RNN) or a single RNN model. On the cloud, additional software components for analysis, re-training and validation of the re-trained models are implemented. Our system is designed to be flexible for using different personalization strategies as and when they become available.

The smartwatch’s UI is designed to start with just the “YES” and “NO” buttons so as to overcome the constraint of small screen space (see Figure 2b). If the user answers “NO” to the question “DID YOU FALL?”, the data is labeled as a false positive and stored as “FP” in the Couchbase database in the cloud. If the user answers “YES”, the subsequent screen will prompt “NEED HELP?”. If the user presses “YES” again, it implies that a true fall is detected and that the user needs help. The collected data will be labeled and stored as “TP” and “HELP IS ON THE WAY” screen will be displayed. If the user presses “NO”, it suggests that no help is needed and the collected data is still labeled as “TP”. If the user did not press either “YES” or “NO” after a specified period of time (seconds displayed in the red circle) following the question “DID YOU FALL?”, an alert message will be sent out automatically to the designated caregiver.

Our system is structured such that all user-identifying data is only stored locally on the phone to preserve privacy. Real-time fall prediction is performed on the phone to reduce the latency of having to send data to the cloud for prediction. The training/re-training of the prediction model is done offline in the cloud server. The UI interface is designed such that there is no need to interact with the App unless the system detects that a fall has occurred, in that case, the watch will vibrate to alert the user that a prediction has occurred and the UI in Figure 2b will appear. The ability to interact with the system when a false prediction is generated allows the system to collect real-world ADL data and fine-tune the fall detection model.

The ultimate goal is for the system to detect falls accurately, i.e., not missing any falls and not generating too many false positive prompts. Collecting data and training a new model from scratch is labor-intensive, hence, we aim to have one model that can generalize well across different smart devices. When a new device is added, by using a small amount of feedback data collected by the user wearing the device for a short period of time, a new model can be trained with a transfer learning strategy and uploaded to the device to use in real time. The following sections describe the transfer learning experiments we conducted to support our vision in this SmartFall system.

## 4. Methodology

### 4.1. Dataset Collection

**Figure 3:**
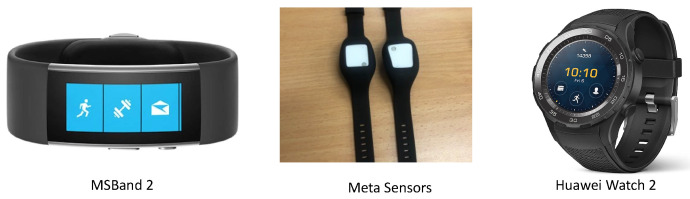
The three different hardware used for data collection.

We first collected three datasets which can be used in the transfer learning experiments. Those datasets are comprised of accelerometer data collected from the Microsoft watch (MSBAND) watch, the Huawei watch, and the meta-sensor device. MSBAND and Huawei data were collected in units of 1G on the left wrist only, while meta-sensor data was collected in units of 2G on both the left and right wrists. The sampling rate is 32 Hz for MSBAND and Huawei watches while meta-sensor data is collected with the sampling rate of 50 Hz. Figure 3 shows the three different devices we used for the data collection process.

The MSBAND dataset was collected from 14 volunteers each wearing a MSBAND watch. These 14 subjects were all of good health and were recruited to perform a mix of simulated falls and ADLs (activity of daily living). Their ages ranged from 21–55, their height ranged from 5 ft to 6.5 ft. and weights from 100 lbs to 230 lbs. Each subject was told to wear the smartwatch on his/her left wrist and perform a predetermined set of ADLs consisting of: walking, sitting down, picking up an object, and waving their hands. This initial set of ADLs were chosen based on the fact there were common activities that involved the movement of the wrists. Those data were all labeled as “NotFall”. We then asked the same subjects to perform four types of falls onto a 12-inch-high mattress on the floor; front, back, left, and right falls. Each subject repeated each type of fall 10 times. We implemented a data collection service on an Android phone (Nexus 5X, 1.8 GHz, Hexa-core processors with 2G of RAM from Google, USA) that paired with the MSBAND smartwatch to have a button that, when pressed, labels data as “Fall” and otherwise “NotFall”. Data was thus labelled in real-time as it was collected by the researcher holding the smartphone. This means when the user was walking towards the mattress before falling down or getting up from each fall, those duration of data will be labelled as “NotFall”. However, the pressing of the button can introduce errors such as the button is being pressed too late, too early, or too long for a fall activity. To mitigate these errors, we post-processed the collected data to ensure that data points related to the critical phase of a fall were labeled as “Fall”. This is done by implementing an R script that will automatically check that for each fall data file, the highest peak of acceleration, and data points before and after that point, were always labeled as “Fall”. After this post-processing of the collected data, we have a total of 528 falls and 6573 ADLs. The MSBAND watch was decommissioned by the vendor in May 2019. This dataset is available http://www.cs.txstate.edu/~hn12/data/SmartFallDataSet.zip, accessed on 3 December 2022.

Huawei watch is compatible with Android WearOS and we designed and implemented an activity labelling app on both the watch and the Android phone for data collection. This activity labeler consists of two components: one on the phone and one on the watch. The watch is paired with the phone using Bluetooth and collects, labels, and sends accelerometer data to the phone in real-time. The phone is considered as a gateway device where labeled data can be stored temporarily and then uploaded to a remote cloud server periodically. The app records accelerometer data sensed from the Huawei watch with a start and stop button, and a user can enter what kind of activity is being recorded before pressing the start button so that the data comes out labeled with a specific activity name rather just “NotFall” as compared with the MSBAND dataset. Twelve students including 7 males and 5 females were asked to perform a prescribed list of ADL activities in triplicate and each type of fall five times. Their ages range from 21 to 35 and their weight average from 100 to 150 lbs. Each participant performed five different types of falls on an air mattress - front, back, right, left, and rotate fall. They were also asked to perform 6 different types of ADL tasks - walking, waving hands, drinking water, wearing a jacket, sitting down, and picking stuff from the floor. Collected data were preprocessed to trim the initial and ending data segment to account for the human errors in pressing or releasing the buttons and to segment the activities and falls into an individual trial for training (since each activity is performed 3 times and each type of fall is performed 5 times). The fall data is further processed into an equal sequence of 100 data points for each fall data sample and a multiple of 100 data points for each ADL data sample. Not all data collected were usable due to missing data points in some falls and ADLs. The final dataset after preprocessing has 144 falls and 271 ADL samples and is available at http://www.cs.txstate.edu/~hn12/data/Huawei_7030.zip, accessed on 3 December 2022.

Meta-sensor was developed by MBIENTLAB in San Francisco ( mbientlab.com, accessed on 3 December 2022). It is a wearable device that offers continuous sensing of motion and environment data. It can sense gyroscopes, accelerometers and magnetometers, and it provides easy-to-use open-source APIs for fast data acquisition. Data can be stored locally on the phone or in a cloud server provided by MBIENLAB. The meta-sensor we used is the MetaMotionRL. The sensor has a weight of 0.2 oz and can be recharged via a USB port. By embedding the meta-sensor in an appropriate wristband, it can serve as a wristwatch for easy collection of ADLs and simulated fall data. The collected data can be exported into multiple file formats. We recruited 8 participants (3 male and 5 female) ages from 22 to 62 for data collection. Each participant was asked to perform four types of falls (front, back, left, and right), five times each on an air mattress, and a prescribed list of ADLs as in the Huawei watch data collection session. These are walking, waving a hand, drinking water, wearing a jacket, sitting down, and picking stuff up from the floor.

The meta-sensor fall data was first programmatically labeled by a Python script that identifies a set amount of peak magnitudes based on the number of trials per file and a uniform width of 35 data points (1.12 s) per fall. Plotting programmatically labelled meta-sensor data in Microsoft Excel showed that labels were often placed around peaks caused by noise rather than actual falls and did not capture the distinct pre-fall, fall, and post-fall activity that accompanied an actual fall. To ensure that we have a set of accurately labeled meta-sensor data to experiment with, we decided to manually relabel all meta-sensor data using Excel plots as a basis for fall window placement. We choose fall windows with a width of 100 data points in an attempt to capture both pre-fall and post-fall activities. To minimize noise, we trimmed non-fall data in between each fall. Since an ADL activity could last much longer than a fall, we label the non-fall data in ADL files to the smallest multiple of 100 data points per trial that could capture the entire activity being performed. The collected meta-sensor data has 202 falls and 492 ADL samples and is available at  http://www.cs.txstate.edu/~hn12/data/Meta_sensor_7030.zip, accessed on 3 December 2022.

### 4.2. Experimental Settings

Transfer learning is a research subject in machine learning that is concerned with the transfer of knowledge obtained while training a model for a specific task, and applying that knowledge as a base model to a different but related task  [18]. For ease of understanding, we select one of our experiments to explain how the transfer learning strategy works in this study. Initially, we use the MSBAND dataset, which we call the source dataset, to train a model from scratch, in turn giving us our preliminary knowledge in the shape of a model that is fully trained to solve the fall detection problem on data sensed by the MSBAND. After that, we use that model as a base model for the meta-sensor dataset, which we call the target dataset, by freezing all of its precursory layers, effectively keeping the weights that resulted from the MSBAND dataset training process as is, and re-training only the dense layers of the model on the meta-sensor dataset. The intuition behind it comes from the small size of the retraining dataset, as the base model resulted from training on a bigger, more complete dataset, making it more desirable in its complex, initial layers, while at the same time transferring over the knowledge needed to normalize the data in the dense layers with respect to the differences between the two datasets. The full transfer learning process is described in Algorithm 1. In the algorithm, we have the source and target datasets as the input. We start off by organizing the data into windows (data windows are explained in Section 4.3) and initializing two models, one suffixed with TFS (Training From Scratch), and the other is suffixed with TL (Transfer Learning). We train the TL model on the full source dataset and freeze its precursory layers, and then evaluate the TL and TFS models on the target dataset by conducting experiments described in Section 5, and compare the performance of the two models in those experiments. All our experiments are conducted on a Dell Precision 7820 Tower, 256 GB RAM from Dell, USA and one GeForce GTX 1080 GPU from Nividia, USA using TensorFlow.
**Algorithm 1:** Our Transfer Learning Structure**Input:** Source Domain Data ***Source_Data***, Target Domain Data ***Target_Data***
Organize ***Source_Data*** And ***Target_Data*** Into Data Windows
Initialize Models ***NN_TL*** And ***NN_TFS***
Train ***NN_TL*** On ***Source_Data*** Data Windows
Freeze ***NN_TL***’s Precursory Layers
Evaluate ***NN_TL*** And ***NN_TFS*** On ***Target_Data*** Data Windows
Compare The Evaluation Results Of ***NN_TL*** And ***NN_TFS*** On ***Target_Data***


### 4.3. Model Training and Parameters Tuning

As mentioned before, we used a simple LSTM neural network structure for our model, as not only does that fit the time-series task well, but it is also a viable option for real-time classification that operates on the edge device without having the need to communicate to the cloud. Our classifier had many different hyperparameters, as well as different options for layer structuring, all of which needed extensive tuning in order to find which permutation of these hyperparameters and structures gives the best result. The main hyperparameters for our classifier are:

- **Window_Size:** The number of consecutive data entries that will be fed to the LSTM classifier at once. For example, if the window size is 35 (meaning the length of a single input block is 35 time-consecutive data entries), then the classifier will be fed a tensor of the shape 35 × 3 (since we have 3 coordinates for acceleration for each entry) to give a single classification for. This snapshot of a particular window size represents one sample of time series data as shown in Figure 1a.
- **Step_Size:** The difference between two consecutive data **blocks** (each block comprised of Window_Size data entries). For example, say we have 37 data entries, with a Window_Size of 35 and a Step_Size of 1, then, we would have 3 different data blocks, them being [1, 35], [2, 36] and [3, 37], which means we have an overlap of 34 entries between each 2 consecutive data entries. If Step_Size was 2, then we would have 2 different data blocks, them being [1, 35] and [3, 37] (the middle block would be skipped since our step is 2), with an overlap of 33 entries between every 2 consecutive entries (Window_Size − Step_Size is the general number of overlapping entries).
- **Smooth_Window:** The way we have our model make a final prediction is by predicting over the last Smooth_Window: data blocks, and then average (take the median of) the predictions and use that average as the final fall probability. The motivation behind the smooth window is to take into account a wider scope of predictions, better covering pre-fall, and post-fall data points. This will also ensure that we do not miss any clustered spikes related to fall and we do not just take a single spike as a fall prediction.
- **Fall Threshold:** After having the averaged fall probability from the most recent smooth window, if its value is greater than Fall Threshold:, then we classify the window as a fall, otherwise we classify it as a non-fall.

As mentioned above, the hyperparameter tuning process needed an extensive amount of experimentation, and for each hyperparameter, we tried a multitude of different numbers from lower to higher values. In this part of the sub-section, we will be describing the experimentation process for each hyperparameter and mentioning what the optimal value is with the reasoning behind it. The hyperparameter turning process was validated on the MSBAND and meta-sensor datasets, for each dataset separately, by splitting that dataset into a training set, which consisted of 70% of the data, and a test/validation set, which consisted of 30% of the data. For each choice of hyperparameters, we would train our classifier on the training set and then calculate the **F1 score** of the trained model on the test set. In the results tables, we show the scores of 5 different values as the other values’ results were similar to the value closest to them in the table.

- **Window_Size:** We tried a multitude of different values, and found that the optimal value is the same as the number of data entries sensed within 1 second (the duration of a fall), meaning that the optimal value for the MSBAND model was 32, as the MSBand is at 32 Hz, and the optimal value for the meta-sensor model was 50, as the meta-sensor is at 50 Hz. This seemed to be the sweet spot that captures enough data for accurate classification, any value below that gave a worse classification accuracy, and any value beyond that did not increase the classification accuracy by a noticeable amount. F1-scores for the different Window_Size values can be found in Table 1, where the optimal values are in bold.
- Step_Size: Out of all the values, a step of 1 seemed to perform the best, which indicates that high overlap and small increments between the consecutive data blocks is important for a good performance, as all the higher values gave worse results. F1-scores for the different Step_Size values can be found in Table 2, where the optimal values are in bold.
- Smooth_Window: As explained before, we want to capture the notion of both pre-fall and post-fall occurrence in order to help us better classify falls and have less false positives, and exactly matching that intuition, a broader smooth window of about 2 seconds of sensed data entries (64 for MSBand and 100 for meta-sensor) out-performed both shorter and longer smooth windows. F1-scores for the different Smooth_Window values can be found in Table 3, where the optimal values are in bold.
- Fall_Threshold: Different values in increments of 10% were tried, starting from 10% and ending at 90%, and the fall threshold of 40% performed the best as it had the best balance of accurate true-positive classification while avoiding as many false-positives as possible. This value wasn’t picked solely through experimentation, but also by looking at the prediction probability of the classifier over the test set, we can see that for the fall data, the classifier predicts values above 40%, and for non-fall data, it predicts values below 40%. F1-scores for the different Fall_Threshold values can be found in Table 4, where the optimal values are in bold.

**Figure 4:**
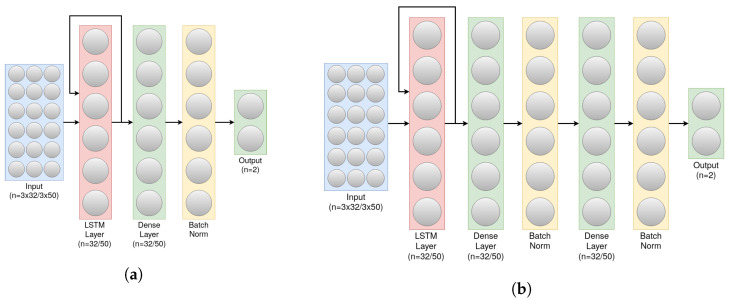
(**a**) Overview of the old classifier’s architecture. (**b**) Overview of an improved LSTM classifier.

As we have mentioned, not only did we tune the hyperparameters of the network, but we also tried several structures for the network itself, mainly following the LSTM layer, as a part of our model tuning. The previous work’s benchmark model is illustrated in Figure 4a.

As we can see, the model consisted of an LSTM layer, followed by a dense layer, batch normalization and ended off with another dense layer. It worked well as is, however, through examining the training accuracy during the training process, the accuracy value seemed to plateau earlier than desired, which is what led to experimenting with the network structure by adding more, but not too many, additional dense layers, up to a point where it would not impact the classification time, and enough to be able to overcome the training accuracy plateau as well as achieve better test accuracy. Indeed, after thorough experimentation, a more optimal structure was achieved, one that had more parameters (from 13,601 to 16,351 parameters), hence more potential for knowledge gain, while maintaining relatively quick classification speed. The new structure simply had 2 additional layers, a batch normalization layer followed by a dense layer. The structure of the new model can be seen in Figure 4b. It is worth noting a few things that are consistent between our model and the previous work’s model:

- All layers are fully connected; using drop-out/convolution layers made the performance of the model slightly worse, hence, why we do not use any of those layers.
- The activation function of the dense layers is Relu, and the last layer uses sigmoid which is commonly used for binary classification.
- The default Keras Library’s Binary Cross-Entropy loss function as well as the default Adam optimizer were used as the loss function and optimizer of the network, as those two worked well in our older version of classifier.
- The number of neurons in the LSTM layer, as well as the output dimensions of the dense layers were always set to the number of data entries sensed in one second, similarly to **Window_Size**, as that generally gave the best result.

## 5. Experiments and Results

In this section, we present our experimental results on transfer learning between the several datasets we described above, the MSBAND dataset, the meta-sensor dataset, and the Huawei dataset. We conduct two main experiments across each pair of datasets. In one of the experiments, we have a source dataset and a target dataset. We start off by building a model from scratch on the target’s training dataset and then testing out that model’s performance on the target’s test dataset. We then build a model using the source’s complete dataset, and then use that model as a base model for the target’s training dataset, test it out on the target’s test dataset, and compare the performance of the two results. In the second experiment, we split the target dataset such that each person’s data is in one data fold, meaning that if we have n different people who volunteered to collect data for a specific dataset, we would split that dataset into n different folds, and conduct a cross-validation on those folds, the first cross-validation being from scratch, and the second cross-validation having the source’s model as a base model for each iteration. This form of leave-one-out cross-validation is more rigorous when the dataset is small. The models’ structures throughout our experiments will all be the exact optimal structure described in the previous section in Figure 4b, as that structure, as explained, performed the best across all three different datasets, while each dataset’s hyperparameters will be specific to that dataset’s smart watch’s hardware specifications, as detailed in Section 4.3.

### 5.1. Left Wrist to Right Wrist Transfer Learning with Meta-Sensor

Our first set of experiments involved purely the meta-sensor dataset, as we wanted to test out the effect of transfer learning when the sensing models share identical hardware specifications, but are, however, applied to different wrists. We started off by building a left-wrist fall detection model, training it from scratch using the left-wrist meta-sensor dataset, using the optimal network structure and hyperparameters choice, which resulted in a fall detection model tailored specifically for the left wrist. Then, using that model, we conducted two different experiments in order to evaluate the effect of transfer learning in the manner described at the beginning of Section 5, which we detail more thoroughly below.

**Figure 5:**
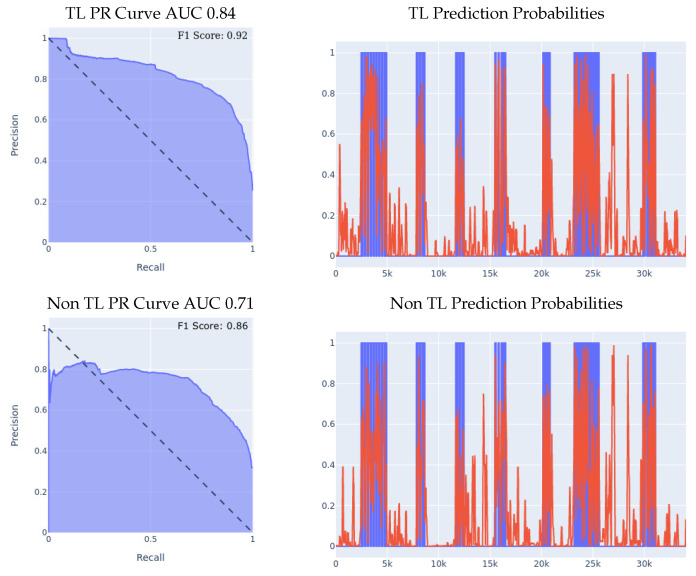
70/30 Train/Test data split experiment for meta-sensor. Note that, **TL stands for Transfer Learning**, for prediction probabilities, *x* axis is the time, *y* axis is the prediction threshold, blue data is the real labels, red data is the prediction probabilities.

**Figure 6:**
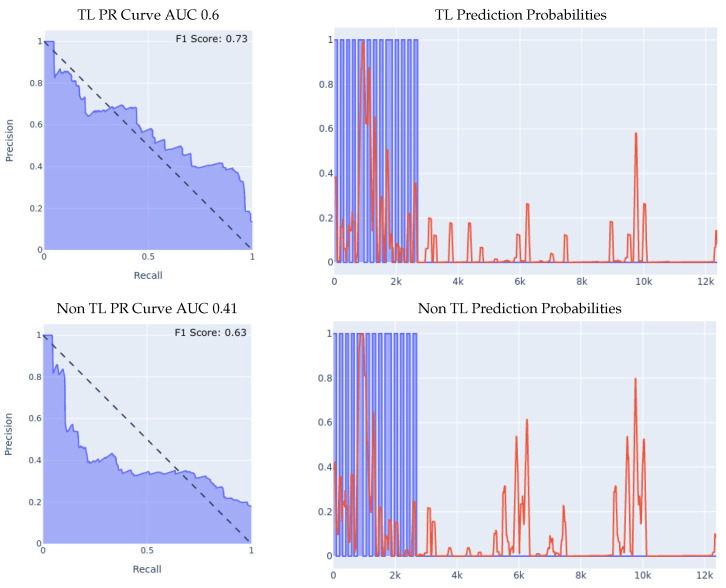
Leave-one-person-out data split experiment for meta-sensor. For prediction probabilities, *x* axis is the time, *y* axis is the prediction threshold, blue data is the real labels, red data is the prediction probabilities. For the F1 Scores, the averaged F1 score of all the 8 iterations of the cross-validation is shown in the top right corner of the PR curve.

1. **Meta-sensor Experiment I:** In the first of the two experiments, we split the right wrist’s dataset into two sets, one of them being a training dataset comprised of 70% of all the data, and the remaining 30% is the test dataset. The content of the two datasets was such that for each of the 8 people in the full dataset, 70% of that person’s data was in the training set, and the remaining 30% was in the test set, which means that this experiment’s main goal is to try and evaluate how well does the model personalize to these specific 8 people after seeing a portion of their data during the training process. After splitting the data in the described manner, we built two different classifiers using the right wrist training data, the first of which was built from scratch using the right wrist training dataset only. The second classifier was built using transfer learning by having the left wrist classifier as a base model and then training that base model on the right wrist training dataset. Results are presented in Figure 5. We can clearly see the effectiveness of transfer learning over building a model from scratch throughout all 3 presented metrics. If we look at the PR curve, we can see that the transfer learning model’s PR curve is more complete and covers more area resulting in a higher AUC. We then evaluated both classifiers’ performance on the right wrist test dataset.
  If we look at the prediction probabilities plot, we can see similar true positive classifications between the two models (keep in mind that the prediction threshold for a fall is 0.4), however, we can also see that the transfer learning model has fewer false positive classification instances, for example, if we look at the entries from 12k to 15k in the x-axis, we can see that the non-transfer learning model predicted them falsely as falls (the real label is in blue, the predicted value is in red, a red value higher than 0.4 means a fall prediction), while the transfer learning model predicted them correctly as non-falls.Finally, if we look at the F1 scores, we can see that the transfer learning model achieved an F1 score that is higher by 8% than the non-transfer learning model as shown in Table 5.
2. **Meta-sensor Experiment II:** In this experiment, we conducted what we call leave-one-person-out cross-validation, which, as its name suggests, is a cross-validation method in which, for each person involved in the meta-sensor dataset, we train the model either from scratch, or using the transfer learning methodology, on a dataset that is comprised of all the people but the one specific person, and then test the resulting model on the remaining person’s data. As mentioned, we do this process for each of the 8 people involved in the full meta-sensor dataset. As opposed to the first experiment, when testing a model in this experiment, the model would have not trained on any data of the person it is being tested on.
  The result of training and testing using a leave-one-out strategy is shown in Figure 6. The PR Curve and Prediction plots are taken from a random iteration of the cross-validation process and are representative of the average iteration. The evaluation results of a single iteration are based on a dataset of one person only, hence the number of data entries in the leave-one-person-out cross-evaluation results are always significantly less than the prior 70/30 Train/Test experiment, as the evaluation results in that experiment are on 30% of the entire dataset. Again, we can clearly see the effectiveness of transfer learning over building a model from scratch throughout all 3 presented metrics. If we look at the PR curve, we can see that the transfer learning model’s PR curve is more complete and covers more area resulting in a higher AUC, even though both models do not achieve the best result, however, the improvement from using transfer learning is substantial, as it made the PR curve over half of the area, while in the non-transfer learning case, it covered less. If we look at the prediction probabilities plot, we can see similar true positive classifications between the two models with the transfer learning model being slightly better, and we can see that the non-transfer learning model has many more prediction peaks and much sharper spikes in the non-fall area, resulting in more false positive predictions. Finally, if we look at the F1 Scores, we can see that the transfer learning model achieved an averaged F1 score that is higher by almost 10% than the non-transfer learning model as shown in Table 5.

### 5.2. MSBAND to Meta-Sensor/Huawei Transfer Learning

Our second set of experiments involved two different inter-device transfer learning experiments. As the main thing we want to test out in our experiments is the effect of transfer learning on small dataset problems, the source of the transfer learning process, aka the base model, is built from training on the Microsoft band dataset, as the MSBAND dataset is the biggest and most complete dataset out of the three, while the meta-sensor dataset, as well as the Huawei dataset, are both smaller in size and in fall samples.

#### 5.2.1. MSBAND to Meta-Sensor

As described above, we started off by training a fall detection model from scratch, using the optimal network structure and hyperparameters choice, on the MSBAND dataset, which resulted in a fall detection model tailored specifically for the MSBAND device, and then, using that model, we conducted two different experiments on the **left** wrist meta-sensor dataset similarly to what we did in Section 5.1.

**Figure 7:**
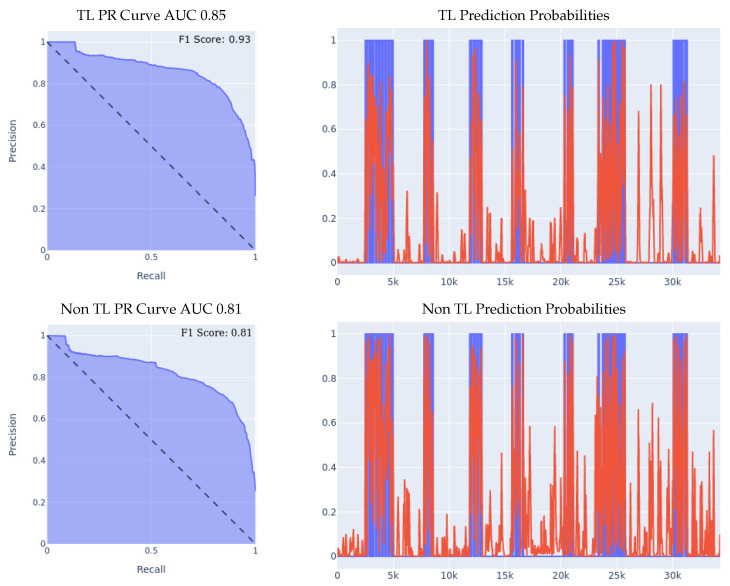
70/30 Train/Test data split experiment for MSBAND to meta-sensor. For prediction probabilities, *x* axis is the time, *y* axis is the prediction threshold, blue data is the real labels, and red data is the prediction probabilities.

**Figure 8:**
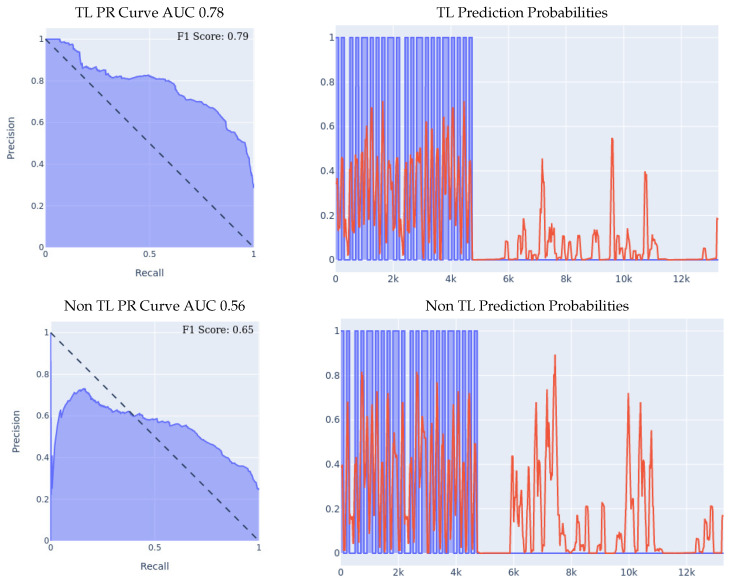
Leave-one-person-out data split experiment for MSBAND to meta-sensor. For prediction probabilities, the *x* axis is the time, the *y* axis is the prediction threshold, the blue data is the real labels, and the red data is the prediction probabilities. For the F1 Scores, the averaged F1 score of all 8 iterations of the cross-validation is shown in the top right corner of the PR curve.

1. **MSBAND to Meta-Sensor Experiment I:** In this experiment, we conduct the exact same 70/30 Train/Test split experiment as we did in the first experiment of Section 5.1. The classifiers’ performance on the left wrist dataset is shown in Figure 7.
  We can see the effectiveness of transfer learning over building a model from scratch throughout all 3 presented metrics. If we look at the PR curve, we can see that the transfer learning model’s PR curve is slightly more complete and covers more area resulting in a higher AUC. If we look at the prediction probabilities plot, we can see that the transfer learning model has fewer false positive classification instances, for example, if we look at the entries from 13k all the way up to 23k in the *x* axis, we can see that the non-transfer learning model predicted a lot of the non-fall entries as falls, while the transfer learning model predicted them correctly as non-fall, resulting in a much lower false positive rate. Finally, if we look at the F1 Scores, we can see that the transfer learning model achieved an F1 score that is higher by 12% than the non-transfer learning model, breaking into the 90% range as shown in Table 5.
2. **MSBAND to Meta-Sensor Experiment II:** We conduct the exact same leave-one-person-out cross-validation experiment as we did in the second experiment of Section 5.1 with the MSBAND and left meta-sensor datasets.
  We compare the results of the two models as shown in Figure 8. The results we obtained show an even higher gap between the transfer learning model and the non-transfer learning model than the experiment we reported in Section 5.1. Again, we can clearly see the effectiveness of transfer learning over building a model from scratch throughout all 3 presented metrics. If we look at the prediction probabilities plot, we can see that the non-transfer learning model has many more prediction peaks and much sharper spikes in the non-fall area, resulting in more false positive predictions in the non-transfer learning case. The F1 Scores with the transfer learning are higher by over 14% than the non-transfer learning model in this experiment as shown in Table 5.

#### 5.2.2. MSBAND to Huawei

We conducted three experiments on the Huawei dataset, the first two experiments being the 70/30 Train/Test split and the leave-one-person-out experiments described in Section 5.1, and the third experiment is a real-time test of the transfer-learning model by one lab volunteer. The real-time test involves wearing the Huawei watch running the SmartFall App describes in Section 3 using a model trained with and without transfer learning.

**Figure 9:**
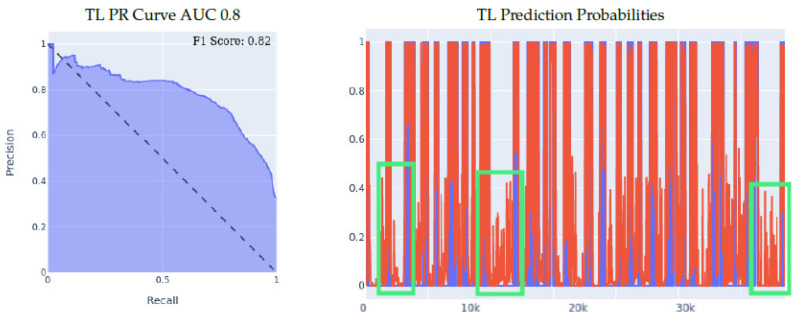

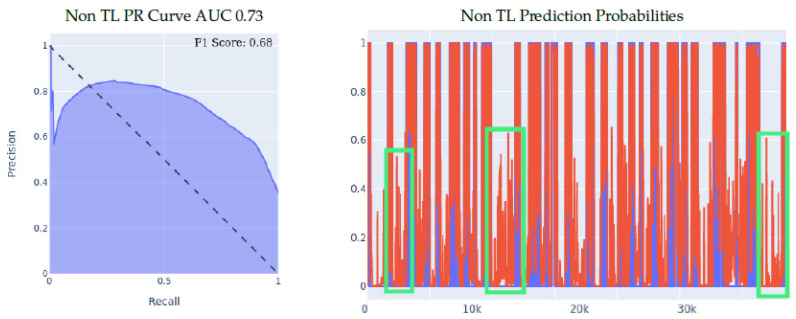
70/30 Train/Test Data Split Experiment for MSBAND to Huawei. For prediction probabilities, the *x* axis is the time, the *y* axis is the prediction threshold, blue data is the real labels, and red data is the prediction probabilities. The green box highlights the areas which contribute to the greatest differences between the two models.

**Figure 10:**
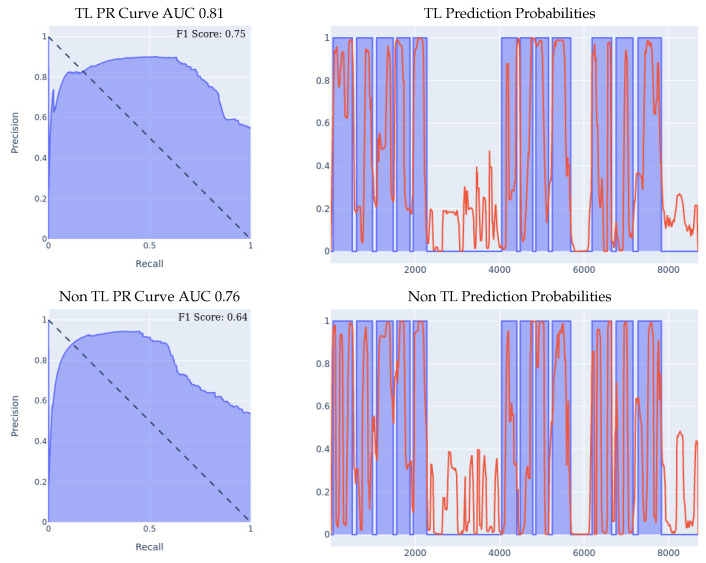
Leave-one-person-out data split experiment for MSBAND to Huawei. The averaged F1 score of all 11 iterations of the cross-validation is shown in the top right corner of the PR curve.

**Figure 11:**
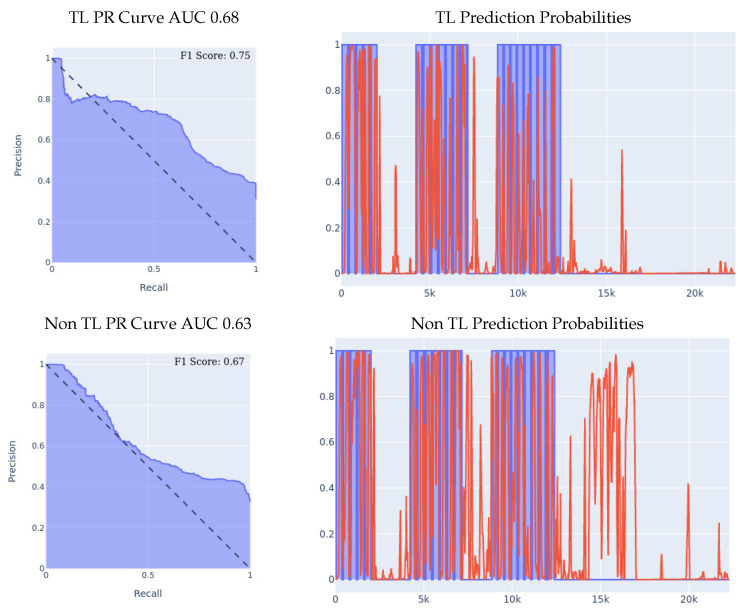
Real-life test experiment. For prediction probabilities, the *x* axis is the time, the *y* axis is the prediction threshold, the blue data is the real labels, and the red data is the prediction probabilities.

1. **MSBAND to Huawei Experiment I:** the results of the 70/30 Train/Test experiment are presented in Figure 9. We can see the effectiveness of transfer learning over building a model from scratch throughout all three presented metrics.
  If we look at the prediction probabilities plot, we can see that the transfer learning model has fewer false positive classification instances, for example, if we look at the entries from 12k to 15k on the *x* axis, we can see that the transfer learning model has much less false positive predictions. The transfer learning model achieved an F1 score that is higher by 14% than the non-transfer learning model as shown in Table 5. Note that in the transfer learning case, the F1 score is not as high as the AUC might imply, and that is because the F1 score is a metric that is focused on the false positive rate and not on the general accuracy, which is an important metric for our evaluation since false positives are a big limitation for our problem.
2. **MSBAND to Huawei Experiment II:** the results of the leave-one-person-out cross validation experiment are presented in Figure 10. If we look at the prediction probabilities plot, we can see that the transfer learning model has fewer false positive classification instances, for example, from 8k onwards, we can see that the transfer learning model has no false positive predictions, while the non-transfer learning model has two false positives, and even though on the entries from 2k to 4k on the *x* axis, both classifiers have two false positive classifications, the transfer learning classifier’s prediction threshold value (the red line) only starts spiking prior to the fall close to entry 4000, in a sense capturing the pre-fall concept, while the non-transfer learning model spikes all through the non-fall range. Finally, if we look at the F1 Scores, we can see that the transfer learning model achieved an F1 score that is higher by 10% than the non-transfer learning model as shown in Table 5.
3. **MSBAND to Huawei real-time experiment:** in this experiment, we present the results of real-time predictions of the transfer learning model against the trained-from-scratch model on a dataset collected via user feedback by a lab volunteer. The dataset contains 25 falls and a series of ADL tasks. The results of the experiment are presented in Figure 11. The transfer learning model achieves a slightly better PR Curve with a slightly higher AUC. If we look at the prediction probabilities plot, we can see that the transfer learning predictions overall are less aggressive, which results in predicting much fewer false positives as seen in entries 13k onwards; however, we can also see that the non-transfer learning model’s aggressiveness actually makes it cover true positives (specifically in ranges 5k–7k and 9k–12k) slightly better than the transfer learning model, resulting in an F1 score gap of 8% in favor of the transfer learning model as shown in Table 5.

### 5.3. Combined Left and Right Wrist Transfer Learning

In our third set of experiments, we wanted to test out the effect of using both left wrist and right wrist fall detection models at the same time (meaning that a user would be wearing a wearable device on both wrists), as well as the effect of transfer learning has on that experiment. For our base model, once again, we use the model created by training on the MSBAND dataset, for the same reasons described above. The experiment we conducted in this was was only the leave-one-person-out experiment. We did so because for the 70/30 Train/Test data split experiment, we already managed to obtain a very good F1 score (as well as good performance in the other metrics) using only one of the wrists, up to 93% in the best case as shown in Table 5.

As before, we split the data such that for each cross-validation iteration, we train two ensemble classifiers, one of them being the ensemble comprised from the left and right wrist meta-sensor models which train from scratch on seven people’s data, and the second model being the ensemble comprised from the left and right wrist meta-sensor models which train on seven people’s data while having the MSBAND classifier as the base model for both members of the ensemble, and then, we evaluate both ensembles’ performance on the eighth person’s dataset. It is important to note that each member of the ensemble for both classifiers trains only on data specific to its wrist, and that both the training and test dataset are synchronized in time between the left and right wrist, as if a person is wearing two meta-sensor devices, one on each wrist, and testing the ensemble’s fall detection (data was indeed collected by subjects who wore the meta-sensor devices on both wrists at the same time).

**Figure 12:**
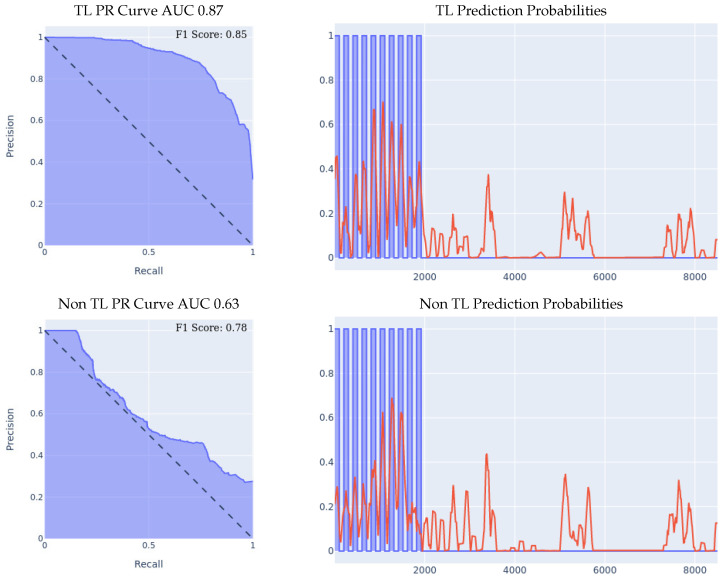
Leave-one-person-out data split experiment for ensemble models. For prediction probabilities, the *x* axis is the time, the *y* axis is the prediction threshold, the blue data is the real labels, and the red data is the prediction probabilities. For the F1 Scores, the averaged F1 score of all the 8 iterations of the cross-validation is shown in the top right corner of the PR curve.

We compare the results of the two ensembles as shown in Figure 12. The PR Curve and Prediction plots are taken from a random iteration of the cross validation process, and are representative of the average iteration. We can see the effectiveness of using an ensemble left and right wrist model over a single wrist model, as well as seeing the effectiveness of transfer learning over building a model from scratch throughout all three presented metrics. If we look at the PR curve, we can see that the transfer learning model’s PR curve is more complete and covers more area resulting in a higher AUC than the normal model. If we also compare both models’ PR curves to the leave-one-person-out experiments detailed in Section 5.1 and Section 5.2, we can see the both models perform better than either of their single wrist counterparts, by having a more complete AUC that covers more area. If we look at the prediction probabilities plot, we can see that the transfer learning ensemble covers more true positives than the ensemble built from scratch (keep in mind that the prediction threshold for a fall is 0.4) while also classifying one less false positive instance. Finally, if we look at the F1 scores, we can see that the transfer learning model achieved an averaged F1 score that is higher by over 7% than the non-transfer learning model, and both of them achieved a higher F1 score than either of their one wrist counterparts, as shown in Table 5. All those experiments results demonstrated the effectiveness of ensemble models using both left and right wrist wearable accelerometers, achieving the best results out of all the models. Such improvements indicated that we can enhance the fall detection prediction by adding more sensors in a scalable way instead of recollecting and re-training a new set of dataset with all the existing sensors.

## 6. Conclusions and Future Work

We presented an approach for fall detection based only on the acceleration data coming from an off-the-shelf wearable edge device on the wrist of the subject. Fall detection using acceleration data coming strictly from a wearable on the wrist is challenging for the reason that there is a lot of room for false positives, as many activities of daily living (ADL) produce acceleration spikes similar to those of a fall. We collected and presented three different types of wearable wrist accelerometers, i.e., the MSBAND smartwatch, the meta-sensor device, and the Huawei smartwatch. Each device has its own hardware specifications, hence making acceleration datasets produced from these three devices differ in many aspects, such as sampling frequency, acceleration unit, axis orientation, etc. Not only are the differences in data between devices a problem, but also, fall data, in general, is very scarce, as it is very time-consuming to collect, leaving us with small datasets across different hardware accelerometers.

In order to overcome the problems detailed above and build a model that is robust to dataset size as well as changes in hardware specifications, we experimented with a transfer learning approach, where we would train a base model from scratch using one device’s dataset, and then use the trained model as a basis for training a new model on a different device’s dataset. Specifically, to solve the target dataset’s task, we would not start training from scratch on the target dataset, but use a model which had already been trained on a source dataset of a similar (but not identical) feature space to the target data set, and then, by training that model on the target dataset and having its weights adapt to the target dataset, we would have effectively transferred the source dataset’s knowledge to the target dataset’s model. We summarized the F-1 score of all the experiments in Table 5.

Indeed, we found out through our experiments, that building a model using transfer learning between different wearable devices produces better results than collecting a new set of data using the device and training a model from scratch, as the former model out-performed the latter in all of the experiments we conducted in the paper. We also experimented with building an ensemble fall detection model using both left and right wrist wearable accelerometers, both from scratch and through transfer learning, and found that both ensemble models out-performed their single-wrist counterparts, with the transfer learning ensemble model achieving the best results out of all the models. This is encouraging as we can improve fall detection by adding more sensors in a scalable way. There is no need to re-collect a new set of datasets with all the existing sensors and re-train everything from scratch when a new sensor is added. We just need to collect a small amount of data using the new sensor and leverage a pre-trained model with transfer learning to generalize to the newly sensed data. We can then combine the final prediction using an ensemble approach.

We have not validated our approach with a target population of different ages, heights, weights, and health conditions. This is a limitation of our current experiment. It is our long-term goal to use part of our funding to recruit older adults for the collection of a small amount of ADL data and use transfer learning for the personalization of fall detection to each person.

One immediate direction for future work is the use of the data augmentation method, for further solving the small training dataset problem. The data augmentation method is a process of artificially increasing the amount of data by generating new data points from existing data that does not require substantial training data, including synthetic minority oversampling technique (SMOTE) [41], transformers [42], auto-Encoder [43], generative adversarial network (GAN) [44]. We have started experimentation with GAN for time series data in [45]. Recently, we have also used a GAN product from Gretel (Gretel.ai, accessed on 25 December 2022) to generate synthetic data. Much more research is needed in this area.

Our second direction is the use of the transfer learning framework for the purpose of personalization for new edge users, as the transfer learning model personalized very well in the 70%/30% Train/Test split experiments. The personalization process can be performed by having a pretrained global model that constantly keeps getting re-trained with newly collected data, and whenever a new user is introduced, we collect a small dataset for that user and train a personalized model specifically for that user through transfer learning from the global model onto the newly collected small dataset.

Finally, we also intend to explore other models, for further improving the accuracy performance of fall detection. Currently, there are many time-series prediction models, such as neural ODEs [46], CT-RNN [47], phased LSTM [48] and transformer [49]. We have just started exploring the transformer model.

## Figures and Tables

**Table 1:** **Window_Size** tuning for MSBAND and meta-sensor datasets, respectively.

MSBAND						Meta-Sensor					
Value	15	20	**32**	40	50	Value	30	40	**50**	60	70
F1-Score	0.8	0.85	**0.93**	0.91	0.92	F1-Score	0.75	0.76	**0.81**	0.81	0.8

**Table 2:** **Step_Size** tuning for MSBAND and meta-sensor datasets, respectively.

MSBAND						Meta-Sensor					
Value	**1**	3	5	7	9	Value	**1**	3	5	7	9
F1-Score	**0.93**	0.9	0.87	0.88	0.86	F1-Score	**0.81**	0.77	0.79	0.75	0.73

**Table 3:** **Smooth_Window** tuning for MSBAND and meta-sensor datasets, respectively.

MSBAND						Meta-Sensor					
Value	20	40	**64**	80	100	Value	20	60	**100**	130	160
F1-Score	0.83	0.89	**0.93**	0.86	0.87	F1-Score	0.69	0.75	**0.81**	0.75	0.78

**Table 4:** **Fall_Threshold** tuning for MSBAND and meta-sensor datasets, respectively.

MSBAND						Meta-Sensor					
Value	0.1	0.3	**0.4**	0.7	0.9	Value	0.1	0.3	**0.4**	0.7	0.9
F1-Score	0.68	0.85	**0.93**	0.81	0.67	F1-Score	0.6	0.76	**0.81**	0.73	0.65

**Table 5:** Summarization results of F1 score for all experiments. Train/Test denotes the train/test dataset split ratio. A check mark represents the transfer learning strategy applied and a × denotes the transfer learning is not applied.

Experiment	Transfer Learning	Dataset Split Strategy	F1 Score (%)
	✓		0.92
meta-sensor Experiment I	×	Train/Test: 70/30	0.86
	✓		0.73
meta-sensor Experiment II	×	cross-validation	0.63
	✓		0.93
MSBAND to meta-sensor Experiment I	×	Train/Test: 70/30	0.81
	✓		0.79
MSBAND to meta-sensor Experiment II	×	cross-validation	0.65
	✓		0.82
MSBAND to Huawei Experiment I	×	Train/Test: 70/30	0.68
	✓		0.75
MSBAND to Huawei Experiment II	×	cross-validation	0.64
	✓		0.75
MSBAND to Huawei real-time experiment	×	100% Test	0.67
	✓		0.85
Combined Left and Right wrist experiment	×	cross-validation	0.78

## Data Availability

Data related to all the transfer learning can be found under Data Collection section of the paper.
